# *Vacuolar protein sorting 35 (Vps35)* rescues locomotor deficits and shortened lifespan in *Drosophila* expressing a Parkinson’s disease mutant of Leucine-rich repeat kinase 2 (*LRRK2*)

**DOI:** 10.1186/1750-1326-9-23

**Published:** 2014-06-11

**Authors:** Radek Linhart, Sarah Anne Wong, Jieyun Cao, Melody Tran, Anne Huynh, Casey Ardrey, Jong Min Park, Christine Hsu, Saher Taha, Rentia Peterson, Shannon Shea, Jason Kurian, Katerina Venderova

**Affiliations:** 1 Department of Physiology and Pharmacology, Thomas J. Long School of Pharmacy and Health Sciences, University of the Pacific, 751 Brookside Rd, Stockton, CA 95211, USA

**Keywords:** Parkinson’s disease, LRRK2, VPS35, Retromer, Endolysosomal pathway, Drosophila, Genetics, Rotenone, Neurodegeneration, Endosomes, Lysosome, VPS26

## Abstract

**Background:**

Parkinson’s disease (PD) is the most common movement neurodegenerative movement disorder. An incomplete understanding of the molecular pathways involved in its pathogenesis impedes the development of effective disease-modifying treatments. To address this gap, we have previously generated a *Drosophila* model of PD that overexpresses PD pathogenic mutant form of the second most common causative gene of PD, Leucine-Rich Repeat Kinase 2 *(LRRK2).*

**Findings:**

We employed this model in a genetic modifier screen and identified a gene that encodes for a core subunit of retromer – a complex essential for the sorting and recycling of specific cargo proteins from endosomes to the trans-Golgi network and cell surface. We present evidence that overexpression of the Vps35 or Vps26 component of the cargo-recognition subunit of the retromer complex ameliorates the pathogenic mutant *LRRK2* eye phenotype. Furthermore, overexpression of *Vps35* or *Vps26* significantly protects from the locomotor deficits observed in mutant *LRRK2* flies, as assessed by the negative geotaxis assay, and rescues their shortened lifespan. Strikingly, overexpressing *Vps35* alone protects from toxicity of rotenone, a neurotoxin commonly used to model parkinsonism, both in terms of lifespan and locomotor activity of the flies, and this protection is sustained and even augmented in the presence of mutant *LRRK2*. Finally, we demonstrate that knocking down expression of *Vps35* in dopaminergic neurons causes a significant locomotor impairment.

**Conclusions:**

From these results we conclude that LRRK2 plays a role in the retromer pathway and that this pathway is involved in PD pathogenesis.

## Background

A growing unmet need for better treatments of neurodegenerative disorders, including Parkinson’s disease (PD), highlights the importance of research into the pathological mechanisms involved in the disease process.

Identification of several causative genes has led to new insights into PD pathogenesis. Leucine-Rich Repeat Kinase 2 *(LRRK2)* (GenBank: AY792511) is the second most common causative gene of PD. Thus far, seven point mutations within *LRRK2* have been demonstrated to segregate with the disease and numerous common and rare *LRRK2* gene variants that increase susceptibility to PD have been described. LRRK2 has also been linked to tau [1] and α-synuclein [2-4] pathologies and therefore may be a key player upstream of cell death pathways involved in other neurodegenerative processes [5]. LRRK2 is a kinase with a Roc-COR catalytic core that has a sequence homology to Rab GTPases. Other domains include LRR and WD-40, predicted to be involved in protein-protein interactions. Despite promising new findings, exactly how LRRK2 contributes to cell death/survival and what is its physiological function, still remains largely unknown.

Although no animal model developed thus far has been able to reproduce all key pathological features of PD, transgenic *Drosophila* models have proven particularly useful, as they faithfully reproduce dopaminergic (DA) neuronal death and locomotor deficits [6-8]. *Drosophila melanogaster* is a highly suitable model organism for studies of gene function, interactions and elucidation of genetic pathways. Notably, *Drosophila* compound eye can be successfully employed in unbiased genome-wide genetic modifier screens *in vivo*[9-11]. Results from such screens and other research in *Drosophila* have recently generated important new insights into the pathophysiology of several neurodegenerative disorders, including PD [12].

To help dissect the molecular processes involved in PD pathology, we recently generated a *Drosophila* overexpressing human *LRRK2* with a PD pathogenic *I2020T* mutation within the kinase domain [13]. This transgenic model has been successfully used by other researchers [14,15]. As shown previously by our team [13] and independently validated by others [16-18], expressing pathogenic mutant *LRRK2* in *Drosophila* DA neurons recapitulates many of the cardinal features of PD, including the loss of DA neurons and locomotor deficits [13]. In addition, *LRRK2* mutant flies present with an abnormal eye phenotype, allowing us to perform an *in vivo* genetic modifier screen in search for genetic interactors of *LRRK2*. Here, we provide evidence that *LRRK2* genetically interacts with *Vacuolar protein sorting 35 (Vps35)* (GenBank: AE013599.4), a core component of the retromer complex.

## Results

### *Vps35* partially rescues the eye phenotype of flies expressing pathogenic mutant *LRRK2*

**Figure 1:**
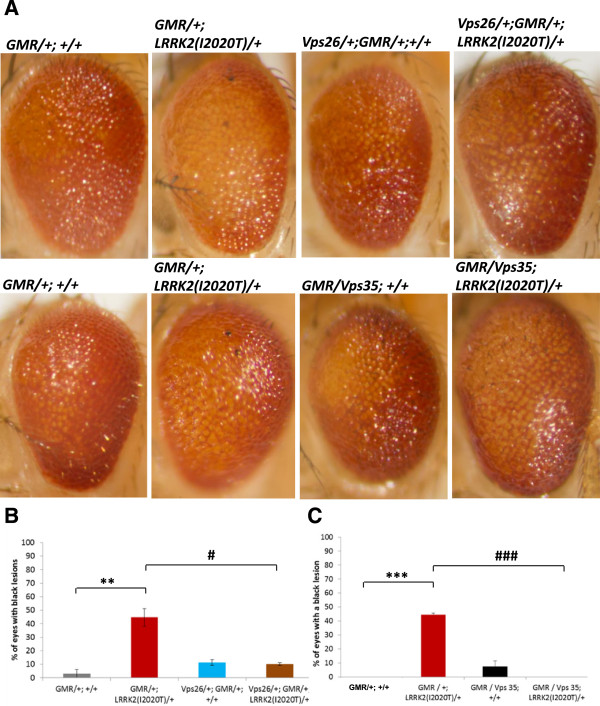
**Eye-specific overexpression of *Vps26 *or *Vps35 *rescues the black lesion phenotype caused by expression of *LRRK2(I2020T). *A)** Representative stereomicroscope images of eyes overexpressing *Vps26* (upper panel) and *Vps35* (lower panel). **B)** Quantification of black lesions in flies overexpressing *Vps26.* Total of 314 eyes from 2-6 independent crosses/genotype (males and females) was analyzed. **C)** Quantification of black lesions in flies overexpressing *Vps35*. Total of 312 eyes from 3 independent crosses/genotype (males and females) was analyzed. All flies were reared at 29°C. Statistical analysis by One-Way ANOVA, Bonferroni’s *post-*test (P‹0.0001). Statistically significant difference compared to control (GMR alone) is denoted as ** for P < 0.001, or *** for P < 0.0001. Statistically significant difference compared to *GMR/+;LRRK2(I2020T)/+i*s denoted as # for P < 0.05, or ### for P < 0.0001.

In our studies, we employed the commonly used *UAS-Gal4* system for a cell/tissue-specific expression [19]. As we have previously shown [13], expression of one of the PD-causing mutants of *LRRK2, LRRK2(I2020T)*, under an eye-specific (*GMR*) promoter at 29°C causes a rough eye phenotype with pigmentation deficits. Notably, 50.03%+/-6.58% of *LRRK2(I2020T)* eyes have black lesions (Figure 1). Similar lesions were reported in other fly models of neurodegeneration [20-24] and seem to be indicative of neuronal (photoreceptor) death occurring later in the eye development, after a full differentiation [21]. Such black lesions are very rare in control (*GMR* alone) flies (3.03%+/-3.03% of eyes) (Figure 1). Employing this *LRRK2(I2020T)* eye phenotype as a read-out in a genetic modifier screen, we identified *Vacuolar protein sorting 26 (Vps26)* (GenBank: NM_130596.2) as a new *LRRK2* interacting gene. Specifically, overexpressing endogenous *Drosophila Vps26* in the eye caused a mild eye phenotype, including an occasional presence of black lesions (11.21% +/- 2.12% of flies) (Figure 1A and B). Strikingly, overexpressing *Vps26* in the *LRRK2(I2020T)* flies rescued the black lesion eye phenotype of the *LRRK2* mutants (10.10% +/- 2.12%; P < 0.05; F(3, 9) = 13.30) (Figure 1A and B).

Vps26, together with Vps35 and Vps29, are the three core components of the cargo-recognition subunit of the retromer complex. Because human homologue of *Vps35, VPS35 (*GenBank: NC_000016.9), has been recently identified as a new candidate PD gene (3, 4), our first goal was to determine whether *LRRK2* also genetically interacts with *Vps35.*

*Drosophila Vps35 (CG5625)* is located on the right arm of the second chromosome and has 61% identity with its human homologue. Again, an eye-specific overexpression of endogenous *Vps35* alone caused a mild eye phenotype that included the occasional presence of black lesions (7.56% of eyes, +/- 3.92%) (Figure 1A and C). Similar to *Vps26*, increased *Vps35* expression completely rescued the black lesion phenotype of *LRRK2* mutants: none of the analyzed eyes displayed any black lesions (P < 0.0001; F (2, 6) = 102.3) (Figure 1A and C). This suggests that both *Vps26* and *Vps35* genetically interact with *LRRK2* in the *Drosophila* eye.

### *Vps35* rescues the locomotor and lifespan deficits of flies expressing pathogenic mutant *LRRK2*

One of the cardinal characteristics of PD is a loss of DA neurons which has a negative impact on movement. Therefore, in our next experiments we used a *Dopa-decarboxylase-Gal4* (*Ddc-Gal4*) driver line that is commonly used in *Drosophila* PD research to target gene expression to DA neurons [6,25].

**Figure 2:**
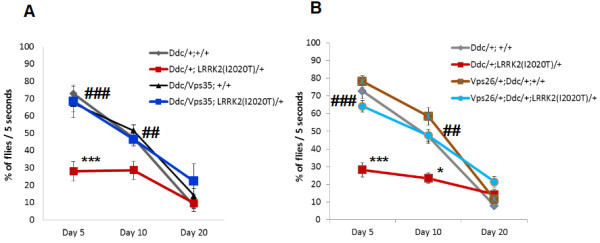
**Overexpression of *Vps26 *or *Vps35 *in DA neurons rescues *LRRK2(I2020T) *locomotor deficits. (A)** Overexpression of *Vps26* in DA neurons. **(B)** Overexpression of *Vps35* in DA neurons. N = 3-12 cohorts of ten per genotype. All flies were reared at 29°C. Statistical analysis: Two-Way ANOVA, Tukey’s *post*-test. Statistically significant difference compared to control (*Ddc* alone) is denoted as * for P < 0.05 and *** for P < 0.0001. Statistically significant difference compared to *Ddc/+;LRRK2(I2020T)* is denoted as ## for P < 0.001 and ### for P < 0.0001.

**Figure 3:**
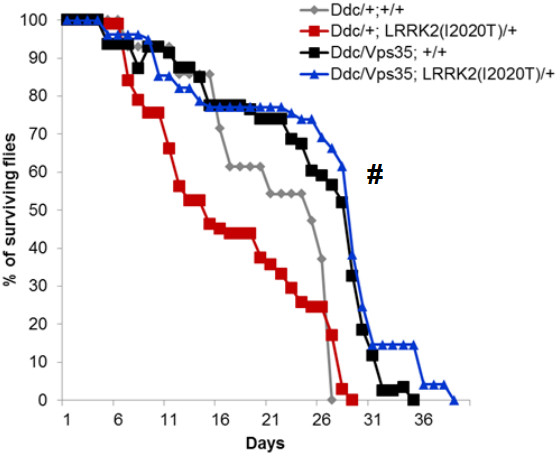
**Overexpression of *Vps35 *in DA neurons rescues the shortened lifespan of *LRRK2(I2020T) *flies.** N = 8-11 cohorts of ten per genotype. All flies were reared at 29°C. Statistical analysis: Two-Way ANOVA, Tukey’s *post*-test. Statistically significant difference compared to *Ddc/+;LRRK2(I2020T)/+i*s denoted as # for P < 0.05.

To quantify locomotor activity, we employed a well-established negative geotaxis climbing assay. As we have shown previously, overexpressing *LRRK2(I2020T)* in DA neurons causes significant locomotor deficits [13]. Compared to control, the climbing ability of *LRRK2(I2020T)* flies was reduced by 61.45% +/- 7.49% on day 5 (specifically, 28.04% +/- 5.45% of *LRRK2* flies were able to reach the line within 5 seconds, compared to 72.75% +/- 7.89% of control) (Figure 2). Similar to the eye, this *LRRK2* phenotype can be fully rescued either by overexpressing *Vps35* in DA neurons (70.00% +/- 6.36% of flies overexpressing *Vps35* and mutant *LRRK2* were able to cross the line within 5 seconds) (P < 0.0001; F (4, 77) = 8.20) (Figure 2B), or by overexpressing *Vps26* (64.12% +/- 3.28% of flies overexpressing *Vps26* and mutant *LRRK2* in DA neurons crossed the line within 5 seconds) (P < 0.0001; F (3, 225) = 11.31) (Figure 2A). Similar to day 5, *Vps35* or *Vps26* overexpression rescued the *LRRK2(I2020T)* phenotype on day 10 (Figure 2A and B). By day 20 however, the locomotor activity of all flies, including control, was severely impaired due to age (Figure 2A and B). Please note that the locomotor activity of flies was always assessed at three different time intervals (5, 10 and 30 seconds), with similar results (data not shown)*.* In addition to the locomotor activity, we also assessed survival of these flies. Compared to the control (*Ddc* alone), survival of flies expressing *LRRK2(I2020T)* in DA neurons was significantly shorter. This phenotype was completely rescued by overexpression of *Vps35* (Figure 3). Altogether, these data validate that mutant *LRRK2(I2020T)* functionally interacts with *Vps35* and *Vps26* in DA neurons and that this interaction is important for the locomotor activity and basal survival of the flies.

### Overexpression of *Vps35* rescues locomotor deficits of other *LRRK2* mutants

The *I2020T* substitution is localized within the kinase domain of *LRRK2*. Our next question was to determine whether the functional interaction between *LRRK2* and components of the retromer complex is specific to this particular mutation. To answer this question, we employed two other transgenic mutant *LRRK2* lines: the *LRRK2(Y1699C)* line carrying a confirmed PD pathogenic mutation in the COR domain of *LRRK2*[26-28], and the *LRRK2(I1122V)* line with a putative pathogenic mutation in the LRR domain [26,27].

**Figure 4:**
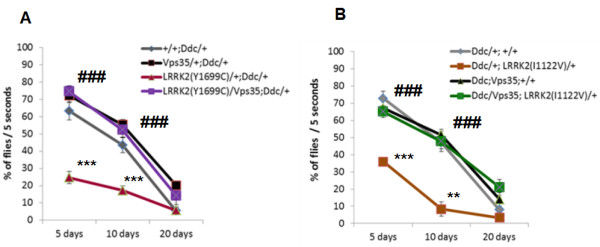
**Overexpression of *Vps35 *in DA neurons rescues locomotor deficits of other PD mutants. (A)** Overexpression of *Vps35* in *LRRK2(Y1699C) mutants.***(B)** Overexpression of *Vps35* in *LRRK2(I1122V) mutants*. All flies were reared at 29°C. N = 3-7 cohorts of ten. Statistical analysis: Two-Way ANOVA, Tukey’s *post*-test. Statistically significant difference compared to control (Ddc alone) is denoted as ** for P < 0.001, or *** for P < 0.0001. Statistically significant difference compared to *Ddc/+;LRRK2(I2020T)/+i*s denoted as ### for P < 0.0001.

Similar to *LRRK2(I2020T),* expressing either one of the two mutant forms of *LRRK2* in DA neurons caused a significant impairment locomotor activity on day 5 (24.71% +/- 3.44% of *LRRK2(Y1699C)* flies were able to reach the line within 5 seconds, compared to 63.21% +/- 5.30% of control, P < 0.0001; F (3, 177) = 50.60; and 39.93% +/- 2.73% of *LRRK2(I1122V)* flies were able to reach the line within 5 seconds, compared to 72.75% +/- 4.27% of control flies, P < 0.0001; F (3, 126) = 23.82, respectively) (Figure 4A and B), which is consistent with our previous results [13]. Importantly, overexpressing *Vps 35* in either one of the two *LRRK2* mutant lines resulted in a complete rescue of the locomotor impairment (74.40% +/-3.42% and 65.01% +/- 3.55%, respectively, of the double transgenic flies were able to reach the top within 5 seconds on day 5; P < 0.0001) (Figure 4A and B). The results were very similar on Day 10 (Figure 4A and B). These results demonstrate that the functional interaction between *LRRK2* and *Vps35* is not exclusive to the kinase domain mutant, and provide further evidence that *LRRK2* may play a role in the retromer-dependent pathway.

### Knocking down components of the cargo-recognition subunit of the retromer complex impairs locomotor activity

*Vps35* is the most recently confirmed causative gene of PD. However the mechanism by which mutation in *Vps35* leads to PD is completely unknown. To better understand the importance of retromer for the physiological function of DA neurons, we analyzed the effect of knocking down expression of genes encoding for components of the retromer complex.

**Figure 5:**
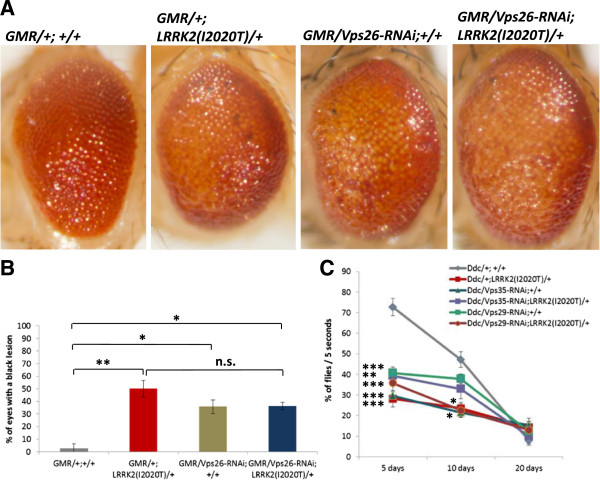
**Knocking down components of the cargo-recognition subunit of the retromer complex causes an eye phenotype and a locomotor impairment.** The phenotypes are not additive with *LRRK2(I2020T)* phenotypes. **A)** Representative stereomicroscope images and **B)** Quantification of black lesions in the eyes *Vps26* knock-down flies*.* Total of 444 eyes from 3-9 independent crosses/genotype (males and females) was analyzed. Statistically significant difference compared to control (*GMR* alone) is denoted as * for P < 0.05 and ** for P < 0.001. Data were statistically analyzed by One-Way ANOVA, Bonferroni’s post-test. **C)** Effect of *Vps35* or *Vps29* knock-down on locomotor activity. N = 3-13 cohorts of ten. Statistical analysis: Two-Way ANOVA, Tukey’s *post*-test. Statistically significant difference compared to control (*Ddc* alone) is denoted as * for P < 0.05, ** for P < 0.001, or *** for P < 0.0001. All flies were reared at 29°C.

First, we analyzed the effect in the eye. Knocking down expression of *Vps26* in the eye caused a significant eye phenotype (35.84% +/- 5.43% of *Vps26* knock-down eyes had a black lesion, compared to 2.78% +/- 3.4% of control eyes; P < 0.05; F (3, 15) = 7.01) (Figure 5A and B). This phenotype was similar to the eye phenotype of the *LRRK2(I2020T)* mutant (44.71 +/- 6.49% of *LRRK2(I2020T)* eyes had black lesions) (Figure 5A and B). Our next goal was to assess whether these two phenotypes are additive. An additive effect would indicate that the two genes likely act on two independent cellular pathways. We observed that the eye phenotypes of *LRRK2(I2020T)* expression and *Vps26* knock-down were not additive (36.21% +/- 3.02% of double transgenic eyes had a black lesion) (Figure 5A and B), suggesting that the two genes act on the same pathway.

The next step was to assess the effect of knocking down components of the retromer complex in DA neurons. In DA neurons, knocking-down expression of *Vps35* or *Vps29* caused a significant impairment in the locomotor activity (29.65% +/- 2.44% and 40.68 +/- 2.93% of flies, respectively, reached the top within 5 seconds on day 5, compared to 72.75% +/- 4.27% of control flies; P < 0.0001, F(5, 320) = 9.335) (Figure 5C). Similar to the effects seen in the eye, these climbing deficits were not additive with those observed in the *LRRK2(I2020T)* mutants (Figure 5C), supporting our results that *Vps35, Vps26* and *Vps29* share a common pathway with *LRRK2.*

### *Vps35* protects from rotenone toxicity

Rotenone is a pesticide and a complex I inhibitor that can be used to model parkinsonism [29]. In our experiments, we exposed the flies to rotenone to assess the role of *LRRK2-Vps35* interaction under conditions of additional cellular stress.

**Figure 6:**
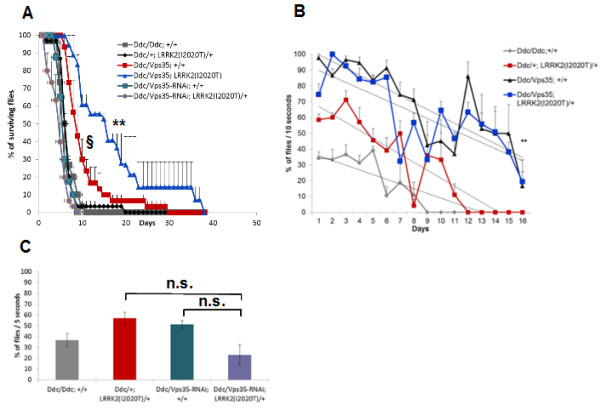
**Effect of *Vps35 *overexpression and *Vps35 *knockdown on rotenone toxicity. A)***Vps35* overexpression in DA neurons improves survival rates following exposure to rotenone, and *LRRK2(I2020T)* and *Vps35* act synergistically to protect from rotenone. **B)***Vps35* overexpression in DA neurons protects from locomotor deficits seen in *LRRK2(I2020T)* or control flies exposed to rotenone. Knocking down Vps35 has no significant effect on survival **(A)**, or locomotor activity **(C)**. N = 20-40 flies/genotype. Statistical analysis by Two-Way ANOVA, Tukey’s *post*-test. Statistically significant difference compared to *Ddc/+;LRRK2(I2020T)* and control (*Ddc* alone) was denoted as * *for P < 0.001; statistically significant difference compared to control and compared to Ddc/Vps35;LRRK2(I2020T)/+was denoted as § for P < 0.05.

Treating control flies with rotenone has a profound effect on their survival. Although we previously showed that pan-neuronal expression of *LRRK2(I2020T)* sensitized flies to low doses of rotenone [13], overexpression of *LRRK2(I2020T)* in DA neurons did not seem to significantly affect survival of flies treated with 1 mM rotenone (Figure 6A). Importantly, overexpressing *Vps35* alone offered a mild but significant protection against rotenone, compared to control flies (Figure 6A). To our surprise however, co-overexpressing *Vps35* and *LRRK2* in DA neurons resulted in a substantial synergistic protective effect against rotenone, demonstrated as a significantly better survival of these flies compared to all other groups (P < 0.0001; F (74, 190) = 3.45) (Figure 6A).

Next, we assessed the climbing ability of the rotenone-treated flies. Rotenone-treated *LRRK2(I2020T)* flies had somewhat unexpectedly a slightly higher locomotor activity compared to control flies (Figure 6B). Again, *Vps35* overexpression alone significantly improved locomotor activity of the rotenone-treated flies (Figure 6B) and this effect was sustained even in the presence of *LRRK2(I2020T)* (P = 0.0011; F (30, 271) = 2.10) (Figure 6B)*.*

Because *Vps35* overexpression protected from rotenone, our next goal was to test whether silencing *Vps35* would sensitize the flies to rotenone toxicity. This would suggest that endogenous *Vps35* is involved in the cellular protection against rotenone. However, our results show that knocking-down *Vps35* had no statistically significant effect on climbing or survival of rotenone-treated flies, compared to control (Figure 6A and C).

Altogether, these data point to a strong functional interaction between *LRRK2* and *Vps35* in DA neurons which may be especially important under conditions of cellular stress. Furthermore, our data for the first time suggest that rotenone interferes with the endolysosomal pathway, although the exact mechanism is not clear.

In summary, we present evidence that overexpression of *Drosophila Vps35,* a core component of the retromer complex, rescues the eye phenotype, locomotor deficits and shortened lifespan of the *LRRK2(I2020T)* expressing flies. Similar to *Vps35*, overexpressing *Vps26* rescued the eye and locomotor phenotypes, thus validating our findings. Moreover, we confirmed that overexpression of *Vps35* also rescues the locomotor phenotypes of two other *LRRK2* mutants. Furthermore, we demonstrate that knocking down *Vps35* leads to a similar degree of locomotor impairment and eye damage as the mutant *LRRK2*, but that the phenotypes of the two genes are not additive. Finally, we show that while silencing *Vps35* has no significant effect on locomotor activity or survival of rotenone-treated flies, overexpressing *Vps35* alone protects from cellular stress caused by rotenone, as demonstrated by prolonging lifespan and improving locomotor deficits, and that this protection by *Vps35* is sustained and even augmented in the presence of mutant *LRRK2.*

## Discussion

Several previous studies have indicated that LRRK2 plays a role in the endolysosomal trafficking [30,31], protein sorting and transport [32] or trafficking of synaptic vesicles [31,33]. For example, overexpression of *LRRK2* is associated with enlarged lysosomes, vacuolization and/or large cytoplasmic punctate structures [30,34,35], suggesting a problem with vesicular trafficking. Here we present evidence that *LRRK2* and *Vps35* functionally interact, and demonstrate how this interaction in DA neurons affects locomotor activity, lifespan and response to rotenone.

*LRRK2* is the most common cause of the monogenic form of PD, and a common risk factor for PD. To further highlight the relevance of our data to PD, the human homologue of *Vps35, VPS35*, has recently been identified as the latest confirmed causative gene of the typical late onset PD, with c.1858 > A (p.Asp620Asn) being the most common *VPS35* mutation [36,37]. This finding has been replicated by several independent analyses [38-42]. However, the mechanism by which VPS35 is involved in PD pathogenesis is entirely unknown.

Our data indicate that *LRRK2* and components of the cargo-recognition subunits of the retromer complex *Vps35* are part of the same molecular pathway, with mutant *LRRK2* likely being upstream and negatively regulating retromer. Furthermore, our gene knock-down data suggest that the mechanism by which the PD pathogenic mutant VPS35 is involved in PD pathogenesis may be a dominant negative mechanism.

VPS35 is a core component of the evolutionarily conserved retromer complex that is predominantly expressed on dynamic endosomal membranes [43-45], to regulate sorting, packaging and directing transport of specific proteins to the trans-Golgi network or cell surface. Thus, the retromer complex prevents specific proteins from being degraded in the lysosome [46]. Retromer consists of two subcomplexes: a cargo recognition subcomplex composed of VPS35, VPS26 and VPS29 [47], and a membrane-interacting subcomplex composed of sorting nexins (SNXs) that bind to a PI3-P-rich endosomal membrane [48]. By regulating protein sorting, retromer is involved in many diverse cellular processes, including but not limited to trafficking of SNARE proteins and receptors such as β-adrenergic [49] or cation-independent mannose 6-phosphate receptors [50], or regulating homeostasis of intracellular glucose, copper and iron.

Although its physiological function in neuronal cells is not yet fully elucidated, it is clear that the retromer-dependent pathway plays a role in etiology or pathophysiology of a number of neurodegenerative processes. For example, genetic variations of *SorLa*[51] or *sorCS1*[52], encoding for receptors that are cargoes for the retromer-dependent pathway, are associated with Alzheimer’s disease, expression of VPS35, VPS26, sortilin and SorLa is altered in Alzheimer’s disease patients [53,54] and interfering with expression of VPS26 or VPS35 [55] causes accumulation of amyloid β and APP derivatives in exosomal compartments [56]. Further research into this pathway may therefore offer important clues and insights into the pathogenesis of PD and other neurodegenerative disorders.

MacLeod *et al.* recently published a paper that demonstrates an interaction between *Vps35* and another *LRRK2* mutant, *LRRK2(G2019S),* where *Vps35* protected against neuronal death caused by *LRRK2(G2019S)*[57]*.* Our data presented here independently validate these findings, and extend the relevance of these findings to two other pathogenic LRRK2 mutants, *LRRK2(I2020T)* and *LRRK2(Y1699C),* and one putative pathogenic mutant *LRRK2(I1122V).* More importantly however, we for the first time characterize the functional role of this interaction in regulation of the locomotor activity, lifespan, sensitivity to rotenone and in retinal degeneration. Furthermore, our data provide evidence that overexpressing *Vps35* alone protects from rotenone, while knocking down *Vps35* has no significant effect on rotenone toxicity. This is consistent with recently published data showing that overexpression of *Vps35* protected *in vitro* against MPP+, another neurotoxin commonly used to model PD, but Vps35 knock-down had no significant effect on cell viability under the same conditions [58].

## Conclusions

In summary, these data provide further evidence in support of the hypothesis that LRRK2 plays a role in the endolysosomal pathway and that the pathology caused by mutant LRRK2 may be at least partly linked to a disruption of this important protein sorting and recycling cellular process. However, how exactly this pathway contributes to PD pathology is at present entirely unknown. Elucidation of new molecular pathways involved in the pathogenesis of PD may bring forward novel pharmacological targets for better treatment strategies. Therefore, a better understanding of the retromer pathway and its relation to PD pathogenesis deserves further investigations.

## Materials and methods

### Drosophila genetics

*UAS-LRRK2(I2020T)*, *UAS-LRRK2(Y1699C)* and *UAS-LRRK2(I1122V)* lines were characterized previously [13]. *P{EPgy2}Vps35*^*EY14200*^*/CyO* and P{EP}Vps26G2008 w*/FM7h flies were obtained from the Bloomington Drosophila Stock Center (BDSC, Indiana University). These lines contain an empty *UAS* element upstream of endogenous *Drosophila Vps35* or *Vps26* gene, respectively, allowing for a *Gal4*-dependent cell/tissue specific gene overexpression [59,60]. *Ddc-Gal4* and *GMR-Gal4* lines were both obtained from BDSC. For the gene knockdown studies, we used the following lines from BDSC: y1 sc* v1; P{TRiP.HMS01858}attP40 (expresses dsRNA for RNAi of Vps35 under *UAS* control); y1 sc* v1; P{TRiP.HMS01877}attP40 (expresses dsRNA for RNAi of CG4764 (FBgn0031310) under *UAS* control), and *y1 v1; P{TRiP.HMS01769}attP40* (expresses dsRNA for RNAi of Vps26 under *UAS* control). To increase transgene expression under the temperature-sensitive *UAS-Gal4* expression system [13], all flies were cultured on a standard cornmeal medium at 29°C (12-hrs dark/light cycle), except for the rotenone-treated flies (see below).

### Eye phenotype

To assess the eye phenotype, we used *GMR-Gal4* to drive the expression of the transgenes in the eye. All crosses and F1 generations were reared at 29°C. At 10 days of age, their eyes were analyzed under a stereomicroscope (Zeiss).

### Negative geotaxis assay and survival assay

The transgenes were overexpressed in DA neurons under a *Ddc* promoter. Progeny of the appropriate genotype were divided into cohorts of ten, and the flies were subjected to a negative geotaxis climbing assay at 5, 10 and 20 days post-eclosure. We recorded and counted flies that crossed a line 8 cm above the base of a transparent tube within 5, 10 and 30 seconds after being gently tapped down. All behavioral experiments were performed at room temperature under standard light conditions. To ensure comparable conditions in each vial, we placed flies in vials with new food every 3-4 days. The same cohorts of flies used in the climbing assay were daily analyzed for survival.

### Rotenone treatment

Cohorts of ten flies (five males and five females) were placed in vials containing freshly prepared rehydrated lyophilized food (Carolina Biological Supplies) containing rotenone (1 mM; Enzo, Farmingdale, NY). Flies were reared at room temperature, their survival and locomotor activity assessed daily, as described above. Every third day, the flies were transferred into a new vial with freshly prepared rotenone-containing food. Because rotenone is light- and temperature-sensitive, the flies were reared at room temperature and in the dark.

### Statistical analyses

All data were analyzed by One-Way ANOVA with a Tukey’s *post-hoc* test, or by Two-way ANOVA followed by a Bonferoni’s *post-hoc* test, as indicated.

## Abbreviations

PD: Parkinson’s disease; LRRK2: Leucine-rich repeat kinase 2; DA: Dopaminergic; Vps35: Vacuolar protein sorting 35; Vps26: Vacuolar protein sorting 26; Vps29: Vacuolar protein sorting 29; BDSC: Bloomington Drosophila Stock Center; Ddc: Dopa-decarboxylase.

## Competing interests

The authors declare that they have no competing interests.

## Authors’ contributions

SAW, JC, MT, AH, CA, JMP, CH, ST, SS, RP and JK participated in carrying out the experiments, RL participated in the design of the experiments, in carrying out the experiments, data analysis and preparation of the manuscript, KV conceived, designed and coordinated the study, participated in carrying out the experiments, analyzed the data and drafted the manuscript. All authors read and approved the final manuscript.
